# Pulmonary arterial stiffening in COPD and its implications for right ventricular remodelling

**DOI:** 10.1007/s00330-018-5346-x

**Published:** 2018-02-27

**Authors:** Jonathan R. Weir-McCall, Patrick SK Liu-Shiu-Cheong, Allan D. Struthers, Brian J. Lipworth, J. Graeme Houston

**Affiliations:** 1 Division of Cardiovascular and Diabetes Medicine, Medical Research Institute, University of Dundee, Dundee, UK; 2 Scottish Centre for Respiratory Research, Medical Research Institute, University of Dundee, Dundee, UK; 3 Division of Cardiovascular and Diabetes Medicine, Ninewells Hospital, Dundee, DD1 9SY UK

**Keywords:** Magnetic resonance imaging, Pulmonary arteries, Heart ventricles, Pulse-wave analysis, Pulmonary disease, chronic obstructive

## Abstract

**Background:**

Pulmonary pulse wave velocity (PWV) allows the non-invasive measurement of pulmonary arterial stiffening, but has not previously been assessed in COPD. The aim of the current study was to assess PWV in COPD and its association with right ventricular (RV) remodelling.

**Methods:**

Fifty-eight participants with COPD underwent pulmonary function tests, 6-min walk test and cardiac MRI, while 21 healthy controls (HCs) underwent cardiac MRI. Thirty-two COPD patients underwent a follow-up MRI to assess for longitudinal changes in RV metrics. Cardiac MRI was used to quantify RV mass, volumes and PWV. Differences in continuous variables between the COPD and HC groups was tested using an independent t-test, and associations between PWV and right ventricular parameters was examined using Pearson’s correlation coefficient.

**Results:**

Those with COPD had reduced pulsatility (COPD (mean±SD):24.88±8.84% vs. HC:30.55±11.28%, *p*=0.021), pulmonary acceleration time (COPD:104.0±22.9ms vs. HC: 128.1±32.2ms, *p*<0.001), higher PWV (COPD:2.62±1.29ms^-1^ vs. HC:1.78±0.72ms^-1^, *p*=0.001), lower RV end diastolic volume (COPD:53.6±11.1ml vs. HC:59.9±13.0ml, *p*=0.037) and RV stroke volume (COPD:31.9±6.9ml/m^2^ vs. HC:37.1±6.2ml/m^2^, *p*=0.003) with no difference in mass (*p*=0.53). PWV was not associated with right ventricular parameters.

**Conclusions:**

While pulmonary vascular remodelling is present in COPD, cardiac remodelling favours reduced filling rather than increased afterload. Treatment of obstructive lung disease may have greater effect on cardiac function than treatment of pulmonary vascular disease in most COPD patients

**Key Points:**

*• Pulmonary pulse wave velocity (PWV) is elevated in COPD.*

*• Pulmonary PWV is not associated with right ventricular remodelling.*

*• Right ventricular remodelling is more in keeping with that of reduced filling.*

**Electronic supplementary material:**

The online version of this article (10.1007/s00330-018-5346-x) contains supplementary material, which is available to authorized users.

## Introduction

COPD is the second most common cause of pulmonary hypertension after left-sided heart disease, with the prevalence of this increasing with increasing severity of COPD [1]. In comparison to other causes of pulmonary hypertension, the pulmonary pressure elevations are modest; however, survival is poor and correlates better with the pulmonary pressures and pulmonary vascular resistance than with the severity of airflow obstruction [2–5].

The role of pulmonary arterial stiffness in COPD was highlighted in a recent study where an increase in pulmonary arterial stiffness was observed during exercise with a greater contribution from this to changes in the mean pulmonary arterial pressures (mPAP) than the peripheral vascular resistance (PVR) [6]. Pulmonary arterial wall thickness is also related to exercise pulmonary pressures rather than resting pulmonary pressures, and correlates highly with change in pressure from rest to exercise [7]. This fits well with observations in idiopathic pulmonary hypertension where a curvilinear relationship exists between RV function and PA distensibility. As a result marked loss of pulmonary artery distensibility occurs without commensurate loss of ventricular function until only minimal distensibility remains when a rapid decompensation of the right ventricle occurs [8]. Thus pulmonary arterial stiffness may be a key early indicator of pulmonary vascular remodelling, and act as a biomarker for future dysfunction before significant adverse remodelling occurs [9].

To date the examination of pulmonary vascular stiffening has been limited due to the need for right heart catheterisation. However, advances in MRI technology have allowed the measurement of pulmonary arterial pulse wave velocity – a direct measure of pulmonary arterial stiffness, which does not require the knowledge of the underlying pulmonary pressures to calculate [10]. Pulmonary PWV has been demonstrated to be feasible, and to be increased in those with pulmonary hypertension; however, it has never been used to examine the pulmonary vasculature in COPD [11–14].

Thus, the aim of the current study was to examine pulmonary arterial PWV in COPD and establish its association with right ventricular remodelling with the hypothesis that: (1) PWV would be elevated early in the disease process of COPD and (2) that elevated PWV would be associated with right ventricular hypertrophic remodelling.

## Materials and methods

Between July 2014 and May 2016 participants were recruited from a database of community spirometry readings, and directly from general practitioner surgeries, clinics and previous research participants. Inclusion criteria for the study were a diagnosis of COPD, based on the current Global Initiative for Chronic Obstructive Lung Disease (GOLD) guidelines of post-bronchodilator forced expiratory volume in 1 s (FEV1)/forced vital capacity (FVC) <0.7 with a history of smoking; and aged 40–85 years. Exclusion criteria were: History of cardiac condition, including but not limited to ischaemic heart disease, valvular disease (mild functional regurgitation allowed), arrhythmia, cardiomyopathy, congestive cardiac failure or congenital cardiac disease; previous cardiac or thoracic operation; other co-existent lung condition; connective tissue disease or systemic vasculitis; severe renal impairment (estimated glomerular filtration rate (eGFR) < 30 ml/min) or contraindication for MRI. All recruited participants underwent a screening echocardiogram to exclude significant silent left ventricular systolic dysfunction (ejection fraction < 45 %).

A healthy control (HC) group was recruited to be approximately age and sex matched, with no prior history of cardiac or pulmonary pathology. All participants gave written informed written consent for the study, which was conducted in accordance with the Declaration of Helsinki and was approved by the East of Scotland Research Ethics Committee 1.

All COPD participants underwent spirometry, DLCO, body plethysmography, a 6-min walk test and a cardiac MRI on the same day. A subset returned at 1 year after their baseline visit for repeat cardiac MRI (CMR). All healthy controls (HCs) underwent a baseline cardiac MRI. Plethysmography and Diffusing Capacity of the Lungs for Carbon Monoxide (DLCO) were performed using a VMax Encore V22 bodybox​ (CareFusion, Basingstoke, UK). These were performed at rest, prior to the 6-min walk test with a minimum of 24 h free from inhaler therapy by dedicated pulmonary function laboratory technicians following ERS/ATS guidelines [15]. The 6-min walk test was performed as per ATS guidelines [16]. The only deviation from these guidelines was the use of a 25-m straight-line course as opposed to a 30-m course.

One hundred and four COPD participants were screened, with 37 excluded due to co-existent coronary artery disease, atrial fibrillation, prior thoracotomy, co-existent lung condition, left ventricular systolic dysfunction on echo or no history of smoking. This left 67 in the cohort. Of these, 58 were included in the final analysis (six excluded due to incomplete scan secondary to claustrophobia, one due to a history of metal fragments in the orbits not picked up in screening and two due to inadequate image quality for analysis). All participants recruited and scanned for the main study before August 2015 went on to have a 1-year follow-up scan. Of the 49 who were scanned prior to this date, 35 underwent their follow-up examination. Sixteen were lost due to: pacemaker insertion (n=1), lung cancer diagnosed on initial CMR and receiving treatment for this (n=2), interval diagnosis of bladder cancer (n=2), unwilling to undergone repeat CMR (n=1), recurrent chest infections (as needed to be free from exacerbations for 2 months) (n=2), withdrawal from the study (n=2), and unable to contact (n=6). Of the 35 scanned, onr had to be abandoned due to claustrophobia despite having successfully completed their CMR the preceding year, and two had images of insufficient quality to analyse, leaving 32 in the final follow-up analysis.

## MRI

Images were acquired with a 3T MR scanner (Prisma, Siemens, Erlangen, Germany). A 32-element cardiac phase-array was used for signal reception. For ventricular quantification a balanced steady-state free precession (bSSFP) stack was performed in breath-hold from the atrioventricular ring to the apex using the following acquisition parameters: Slice thickness 6 mm, interslice gap 4 mm, TR/TE 47.6/1.49 ms, no. averages 1, phases 25, bandwidth/pixel 446 Hz, flip angle 53°, field of view (FOV) 360 × 360 mm^2^, FOV phase 84.4 %, matrix 256 × 256, parallel acceleration factor 2. To plan the main pulmonary artery phase contrast sequence, a bSSFP sequence of the right ventricular outflow tract was performed following which an orthogonal plane was acquired to optimally visualise the main pulmonary artery and pulmonary valve. A breath-hold bSSFP sequence was then performed through the MPA slice and was positioned mid-way between the valve and the bifurcation of the pulmonary artery in order to avoid both structures throughout the cardiac cycle. A free-breathing phase-contrast sequence (slice thickness 6 mm, TR/TE 12/4 ms, no. averages 1, phases 80, velocity encoding 150 cm/s, bandwidth/pixel 340 Hz, flip angle 15°, field of view (FOV) 320×320 mm^2^, matrix 512×512) was then performed in the same position as previously described [17].

### Image analysis

Image analysis was performed using CVI 42 (Circle Cardiovascular Imaging Inc., Calgary, Alberta, Canada).

#### Ventricular quantification

Epicardial and endocardial contours were drawn around the right ventricle at end-systole and end-diastole. Trabeculae were included in the mass measurement and excluded from the volume calculation due to previous work showing that inclusion of the trabecular mass improves correlation with pulmonary pressures and vascular resistance [18]. The septum was treated as belonging to the left ventricle and was excluded from the right ventricular mass. Right ventricular mass and volumes were normalised to height^1.7^. Twenty scans were analysed twice for reproducibility, with excellent reproducibility for all measures (intraclass correlation (ICC) > 0.8 for all; see Supplementary Material for full ICC (Table S1) and Bland-Altman analysis (Fig. S1)).

#### Pulmonary stiffness

**Fig. 1:**
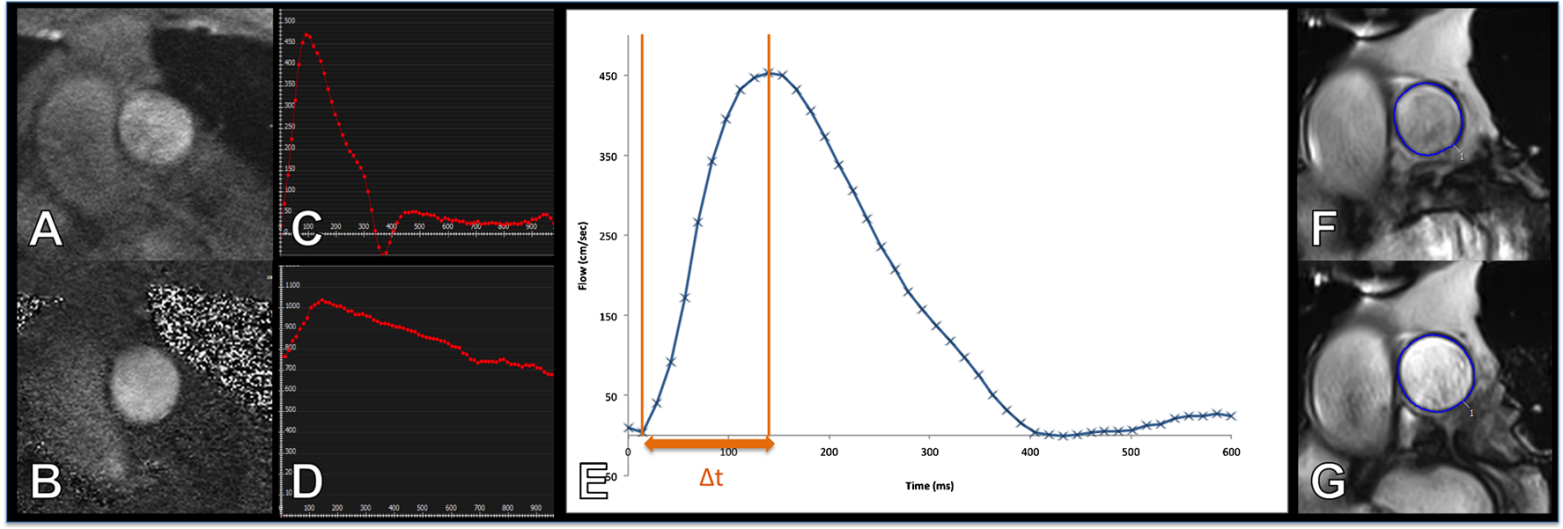
Calculation of pulse wave velocity, pulmonary acceleration time and pulmonary pulsatility. Magnitude (**A**) and phase (**B**) images of the main pulmonary artery from which flow (**C**) and area (**D**) data are acquired from which the pulse wave velocity is derived. A zoomed in period from early systole (**E**) demonstrates the calculation of the pulmonary acceleration time (∆t). Cine images of the main pulmonary artery at end diastole (**F**) and end systole (**G**) are used to calculate pulmonary pulsatility

Pulmonary stiffness was measured using PWV, pulmonary acceleration time (PAT) and pulsatility (see Fig. 1). For PWV, a contour was manually drawn around the perimeter of the vessel on the magnitude image and then propagated throughout the cardiac cycle and corrected when automatic contouring led to erroneous boundaries. The program then automatically calculated area, flow and velocity data, which were exported to Excel 2010 (Microsoft, USA). The area and flow were plotted against one another during early systole, which was defined as the time period in systole during which both the vessel area and flow were simultaneously increasing. The PWV was then calculated as described by Davies et al. [19]:

$$ PWV=\sqrt{\frac{\sum \Delta  {Q}^2}{\sum \Delta  {A}^2}} $$

The reproducibility of this technique has been described in detail previously [17, 20]. Pulmonary acceleration time (PAT) was calculated as the time from first increase in the flow at the start of systole to the point of maximum flow in systole using the phase-contrast sequences. Pulmonary pulsatility was measured from a bSSFP short axis view of the pulmonary artery, which was performed using the same acquisition parameters as the ventricular analysis bSSFP sequences. Pulsatility was calculated as [21]:

$$ Pulsatility=\frac{Maximum\ area- minumum\ area}{Minimum\ area}\times 100 $$

### Statistics

The study was powered to examine the longitudinal effects of PWV on right ventricular remodelling. Due to a lack of prior work on the longitudinal follow-up of ventricular remodelling in COPD, the primary study outcome – change in right ventricular mass – was powered according to the placebo arms of randomised control trials in pulmonary hypertension as this was the only data available. These describe an increase in mass of 5–8 g (change in right ventricular mass detected over 4–36 months in groups of 21 and 44 participants, respectively) [22, 23]. Based on these factors, n=17 would allow detection of a 4-g change with a study power of 80 % and a *p*=0.05. Further work by Bradlow et al. has shown a group size of 17 will allow detection of a 10-ml change in RV end-diastolic volume, which correlates with changes of 9–15 ml seen in the placebo arm of drug intervention studies [22–24].

Descriptive statistics were used for the analysis of the demographic and clinical features of the cohorts with data expressed as mean ± SD. Normality and equality of variances of the variables was tested. An independent sample t-test was used to compare the differences in continuous variables between the HC and COPD cohort. To assess the longitudinal impact of PWV on RV remodelling in COPD, this cohort was divided into three according to the tertile of their baseline PWV, and an independent sample t-test was used to compare the difference in change in the RV mass and volumes between those in the top and bottom tertiles of PWV. Chi-square and Fisher’s exact tests were used as appropriate to compare differences in ordinal and nominal data between the groups. Pearson’s correlation coefficients were used to assess the correlation between PWV and demographic, spirometric and MRI factors. Analysis of covariance was performed to further analyse differences in RV metrics and PWV between the HC and COPD cohorts correcting for differences in baseline demographics. Paired-sample t-tests were used to examine differences in metrics between baseline and follow-up. Reproducibility of measures was examined using a two-way absolute agreement fixed effect ICC and Bland-Altman plots. All data were analysed using SPSS statistical package (version 21.0, SPSS Inc. Chicago, IL, USA). Significance was assumed when *p* < 0.05.

## Results

Fifty-eight COPD patients (67.4±9.0 years old, 55 % male) and 21 HCs (60.4±5.1 years old, 48 % male) completed the study protocol. Despite approximate age and sex matching, those with COPD were significantly older (*p*<0.001), had a higher BMI (COPD: 26.8±5.2 kg/m^2^ vs. HC: 24.7±2.5 kg/m^2^, *p*=0.02), and a higher resting heart rate (COPD: 73.8±20.3 bpm vs. HC: 64.2±11.8 bpm, *p*=0.05). Of those with COPD, the majority had moderate COPD, with n=12 with GOLD I, n=32 with GOLD II, n=13 with GOLD III and n=1 with GOLD IV COPD. Using the modified British Medical Research Council (mMRC) breathlessness score [25], experienced breathlessness was also on the milder end of the spectrum, with n=5 grade 0, n=26 grade 1, n=14 grade 2, n=11 grade 3 and n=2 grade 4. Full baseline characteristics are detailed in Table 1. The 32 who completed the follow-up visit (mean age = 68.6±8.2 years, 50 % male) had a significantly lower heart rate at baseline, but were otherwise no different from the 26 who only underwent baseline imaging (see Table S2).

**Table 1:** Demographics of the COPD and healthy control cohorts

	Healthy controls	COPD cohort	*p*-value
N	21	58	
Age (y)	60.4±5.1	67.5±9.2	**<0.001**
Sex (male)	10 (48 %)	30 (52 %)	0.75
BMI (kg/m^2^)	24.7±2.5	26.8±5.2	**0.02**
Heart rate (bpm)	64.2±11.8	73.8±20.3	0.05
Systolic BP (mm Hg)	127.6±13.9	131.1±20.0	0.47
Diastolic BP (mm Hg)	74.7±7.8	75.1±8.5	0.85
Smoking status			
Current smoker	2 (10 %)	16 (28 %)	**<0.001**
Ex-smoker	7 (33 %)	42 (72 %)	
Never smoker	12 (57 %)	0 (0 %)	
Pack years	4.68±7.4	48.5±24.0	**<0.001**
Medications			
SABA	0 (0 %)	54 (93 %)	<0.001*
SAMA	0 (0 %)	1 (2 %)	1.0*
LABA	0 (0 %)	8 (14 %)	0.10*
LAMA	0 (0 %)	36 (62 %)	<0.001*
ICS	0 (0 %)	7 (12 %)	0.18*
LABA/ICS combo	0 (0 %)	28 (48 %)	<0.001*
Oral steroid	2 (10 %)	4 (7 %)	0.65*
Antibiotics	0 (0 %)	2 (3 %)	1.0*
Theophylline	0 (0 %)	4 (7 %)	0.56*
Mucolytics	0 (0 %)	9 (16 %)	0.10*
GOLD status			
I	-	12 (21 %)	-
II	-	32 (55 %)	-
III	-	13 (22 %)	-
IV	-	1 (2 %)	-
mMRC grade			
0	-	5 (9 %)	-
1	-	26 (45 %)	-
2	-	14 (24 %)	-
3	-	11 (19 %)	-
4	-	2 (3 %)	-

*BMI* body mass index, *BP* blood pressure, *SABA* short-acting beta-agonist , *SAMA* short-acting muscarinic antagonist, *LABA* long-acting ß-agonist, *LAMA* long-acting muscarinic antagonist, *ICS* inhaled corticosteroid, *GOLD* Global Initiative for Chronic Obstructive Lung Disease, *mMRC* Modified British Medical Research Council

*Fisher’s exact test used for between-group analysis

Those with COPD demonstrated evidence of pulmonary arterial stiffening and pulmonary vascular remodelling with higher pulmonary artery area at end diastole (COPD: 2.36±0.56 cm^2^/m^1.7^ vs. HC: 2.14±0.28 cm^2^/m^1.7^, *p*=0.027), reduced pulsatility (COPD: 24.88±8.84 % vs. HC: 30.55±11.28 %, *p*=0.021), reduced PAT (COPD: 104.0±22.9 ms vs. HC: 128.1±32.2 ms, *p*<0.001) and higher PWV (COPD: 2.62±1.29 ms^-1^ vs. HC: 1.78±0.72 ms^-1^, *p*=0.001). Given that the COPD cohort were older with a higher rate of smoking and a higher BMI, an ANCOVA was performed with PWV and these variables. A small-to-moderate group effect size was seen between COPD and HCs for PWV, although this did not quite reach significance (partial eta squared = 0.05, F=4.0, *p*=0.050) (see Table S3). Compared with the HCs, those with COPD had significantly smaller right ventricular end-diastolic volumes (HC: 59.9±13.0ml/m^1.7^ vs. COPD: 53.6±11.1ml/m^1.7^, *p*=0.037), stroke volume (HC: 37.1±6.2ml/m^1.7^ vs. COPD: 31.9±6.9ml/m^1.7^, *p*=0.003) (see Table 2 for full ventricular and pulmonary arterial parameters). These between-group differences persisted on ANCOVA analysis correcting for age, BMI and smoking for RVEDV (F=4.6, *p*=0.035) and RVSV (F=8.8, *p*=0.004) (see Table S3).

**Table 2:** Ventricular quantification and measures of pulmonary arterial stiffness and haemodynamics in the healthy control and COPD cohorts

	Healthy controls	COPD cohort	*p*-value
N	21	58	
Right ventricle			
RVEDV (ml/m^1.7^)	59.9±13.0	53.6±11.1	**0.04**
RVESV (ml/m^1.7^)	22.9±8.6	21.8±7.3	0.58
RVSV (ml/m^1.7^)	37.1±6.2	31.9±6.9	**0.003**
RVEF (%)	63.0±7.9	59.8±7.8	0.10
RVM (g/m^1.7^)	15.1±3.2	16.3±3.4	0.53
RVMVR (g/ml)	0.27±0.04	0.31±0.06	**0.004**
Left ventricle			
LVEDV (ml/m^1.7^)	59.9±9.9	56.1±12.0	0.17
LVESV (ml/m^1.7^)	23.9±6.4	23.3±9.3	0.77
LVSV (ml/m^1.7^)	35.9±5.0	32.9±6.7	0.06
LVEF (%)	60.5±6.0	59.3±8.6	0.57
LVM (g/m^1.7^)	41.4±7.5	43.4±9.4	0.37
Pulmonary artery			
Max area (cm^2^/m^1.7^)	2.80±0.48	2.94±0.74	0.33
Min area (cm^2^/m^1.7^)	2.14±0.28	2.36±0.56	**0.03**
Pulsatility (%)	30.55±11.28	24.88±8.84	**0.02**
PAT (ms)	128.1±32.2	104.0±22.9	**<0.001**
PWV (ms^-1^)	1.78±0.72	2.62±1.29	**0.001**

*LVEDV* left ventricular end-diastolic volume, *LVESV* left ventricular end-systolic volume, *LVSV* left ventricular stroke volume, *LVEF* left ventricular ejection fraction, *LVM* left ventricular mass, *PWV* pulse wave velocity, *PAT* pulmonary acceleration time, *RVEDV* right ventricular end-diastolic volume, *RVESV* right ventricular end-systolic volume, *RVSV* right ventricular stroke volume, *RVEF* right ventricular ejection fraction, *RVM* right ventricular mass, *RVMVR* right ventricular mass:volume ratio

PWV was associated with BMI (R=-0.28, *p*=0.03) and diastolic blood pressure (R=0.35, *p*=0.009), and total lung capacity (R=0.28, *p*=0.039), with a trend towards an association with residual volume (R=0.25, *p*=0.067), but did not demonstrate any significant association with any right ventricular parameters (see Table 3).

**Table 3:** Pearson’s correlation coefficients of pulse wave velocity (PWV) with demographic, spirometric and right ventricular measures

	PWV	
	Rho (95 % CI)	*p*-value
Age	0.04 (-0.24–0.28)	0.75
BMI (kg/m^2^)	**-0.28 (-0.49–0.02)**	**0.03**
Heart rate (bpm)	0.20 (-0.11–0.46)	0.14
Systolic BP (mm Hg)	0.14 (-0.18–0.45)	0.29
Diastolic BP (mm Hg)	**0.35 (0.06–0.60)**	**0.009**
SpO_2_	-0.06 (-0.40–0.27)	0.65
Pack years	0.01 (-0.24–0.26)	0.94
FEV1, % predicted	-0.16 (-0.37–0.09)	0.24
FVC, % predicted	-0.07 (-0.28–0.14)	0.61
FEV1/FVC	-0.18 (-0.42–0.10)	0.18
FEF 25–75, % predicted	-0.11 (-0.35–0.14)	0.40
DLCO, % predicted	-0.05 (-0.28–0.27)	0.73
DLCO/VA, % predicted	-0.07 (-0.33–0.26)	0.60
RLV, % predicted	0.25 (-0.03–0.47)	0.07
VC, % predicted	-0.02 (-0.26–0.23)	0.88
TLC, % predicted	**0.28 (0.00–0.51)**	**0.04**
RLV /TLC	0.21 (-0.08–0.43)	0.13
6MWT (m)	–0.17 (–0.39–0.07)	0.20
RVEDV (ml/m^1.7^)	-0.12 (-0.36–0.18)	0.39
RVESV (ml/m^1.7^)	-0.20 (-0.39–0.08)	0.14
RVSV (ml/m^1.7^)	0.02 (-0.22–0.28)	0.86
RVEF (%)	0.18 (-0.04–0.38)	0.18
RVM (g/m^1.7^)	-0.24 (-0.49–0.02)	0.07

*DLCO* diffusing capacity of the lungs for carbon monoxide, *FEF* forced expiratory flow, *FEV1* forced expiratory volume in 1 s, *FVC* forced vital capacity, *PWV* pulse wave velocity, *PAT* pulmonary acceleration time, *RVEDV* right ventricular end-diastolic volume, *RVESV* right ventricular end-systolic volume, *RVSV* right ventricular stroke volume, *RVEF* right ventricular ejection fraction, *RVM* right ventricular mass, *RLV* residual lung volume, *TLC* total lung capacity, *VC* vital capacity, *6MWT* 6-min walk test

As the pulmonary stiffness did not appear to affect cardiac remodelling and the cardiac remodelling appeared to be that of a reduced preload (with reduced ventricular volumes) rather than an increased afterload (which would be associated with an increase in right ventricular mass) the determinants of the cardiac volumes were examined as detailed in Table 4. RVEDV and LVEDV demonstrated a strong correlation with one another (R=0.64, *p*<0.001). Both RVEDV and LVEDV demonstrated significant correlations with heart rate (RVEDV R=-0.41, *p*=0.04; LVEDV R=-0.30, *p*=0.02) and DLCO (RVEDV R=0.29, *p*=0.03; LVEDV R=0.28, *p*=0.03) and either a trend or a significant correlation with KCO (RVEDV R=0.27, *p*=0.046; LVEDV R=0.25, *p*=0.06) and BMI (RVEDV R=0.27, *p*=0.04; LVEDV R=0.21, *p*=0.11).

**Table 4:** Correlation coefficients between right and left ventricular end-diastolic volumes and demographic, spirometric and pulmonary measures

	RVEDV		LVEDV	
	r	*p*	r	*p*
Age	-0.05	0.69	-0.02	0.99
BMI (kg/m^2^)	**0.27**	**0.04**	0.21	0.11
Heart rate (bpm)	**-0.41**	**0.002**	**-0.30**	**0.02**
Systolic BP (mm Hg)	0.02	0.90	0.14	0.31
Diastolic BP (mm Hg)	-0.13	0.35	-0.07	0.63
SpO_2_ (%)	-0.02	0.91	0.03	0.85
Pack years	0.11	0.41	0.05	0.69
FEV1, % predicted	0.22	0.09	0.18	0.17
FVC, % predicted	0.13	0.35	0.03	0.81
FEV1/FVC	0.21	0.12	-0.11	0.44
FEF 25–75, % predicted	0.23	0.08	0.23	0.08
DLCO, % predicted	**0.29**	**0.03**	**0.28**	**0.03**
KCO, % predicted	**0.27**	**0.046**	0.25	0.06
RLV, % predicted	-0.21	0.12	-0.02	0.91
TLC, % predicted	-0.23	0.10	0.12	0.38
VC, % predicted	0.11	0.44	0.21	0.12
RLV/TLC	-0.20	0.15	-0.17	0.23

*DLCO* diffusing capacity of the lungs for carbon monoxide, *FEF* forced expiratory flow, *FEV1* forced expiratory volume in 1 s, *FVC* forced vital capacity, *PWV* pulse wave velocity, *PAT* pulmonary acceleration time, *RLV* residual lung volume, *TLC* total lung capacity, *VC* vital capacity, *6MWT* 6-min walk test

**Fig. 2:**
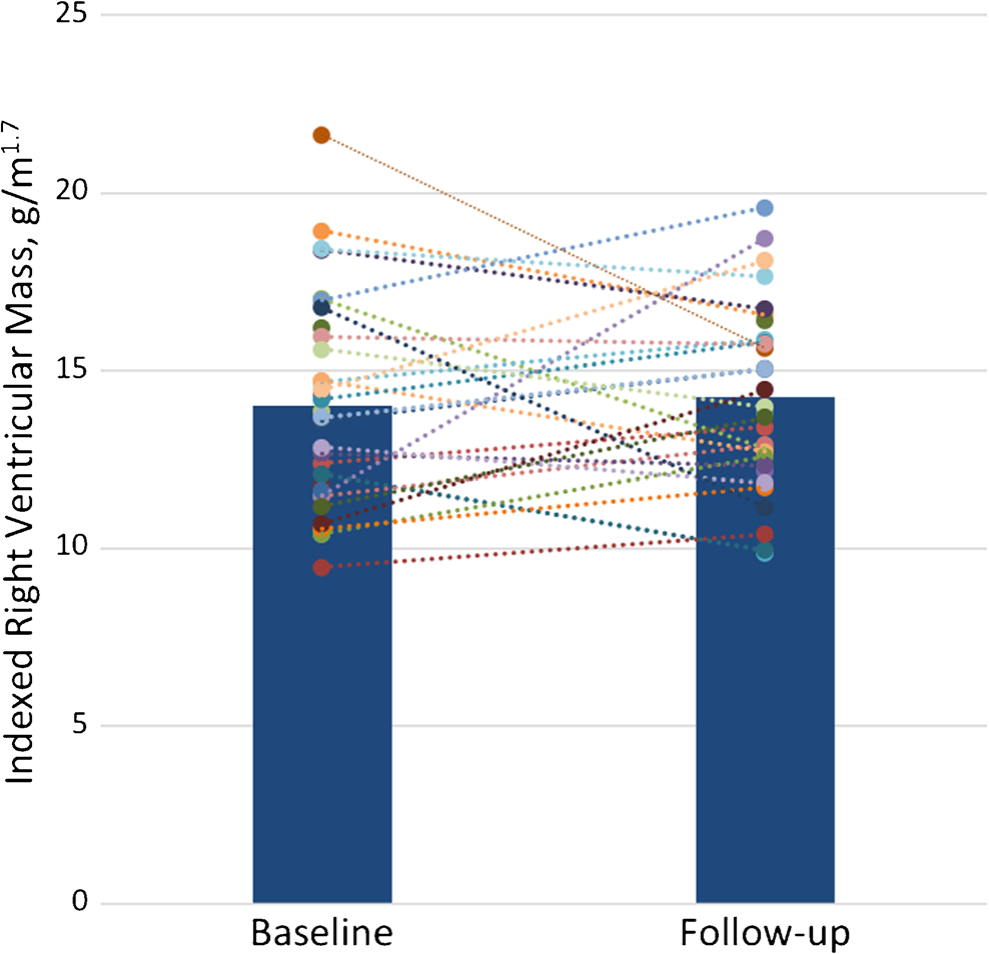
Bar (representing the mean) and dot (representing the individual participants) plot of the change in right ventricular mass from baseline to follow-up examination

At follow-up there was no significant change in right ventricular mass or volumes (see Fig. 2 for changes in RVM and Table S4 for full comparison of follow-up metrics). Those with the stiffest arteries at baseline (upper tertile of PWV) had no significant differences in right ventricular remodelling at follow-up than those with the most elastic arteries (bottom tertile of PWV). For a full comparison of the change in ventricular metrics between PWV tertiles see Table 5.

**Table 5:** Comparison of the change in right ventricular measures at 1-year follow-up across baseline pulse wave velocity (PWV) tertiles

	Tertile			*p*-value*
	1	2	3	
N	10	11	11	
Baseline PWV	1.31±0.26	2.18±0.32	3.42±0.59	
*CMR*				
∆RVEDV (ml/m^1.7^)	-3.9±8.1	-1.6±7.0	1.3±8.9	0.18
∆RVESV (ml/m^1.7^)	-2.5±7.0	0.6±3.7	2.0±5.9	0.13
∆RVSV (ml/m^1.7^)	-1.3±5.6	-2.2±3.9	-0.5±5.9	0.75
∆RVEF (%)	0.7±8.6	-2.2±3.3	-1.5±7.3	0.55
∆RVM (g/m^1.7^)	-0.66±2.8	0.26±4.2	0.53±2.3	0.29
∆RVMVR (g/ml)	0.01±0.07	0.01±0.08	0.02±0.07	0.71

*p-value is tertile 3 (stiffest pulmonary arteries) compared to tertile 1 (most elastic pulmonary arteries)

*PWV* pulse wave velocity, *RVEDV* right ventricular end-diastolic volume, *RVESV* right ventricular end-systolic volume, *RVSV* right ventricular stroke volume, *RVEF* right ventricular ejection fraction, *RVM* right ventricular mass, *RVMVR* right ventricular mass:volume ratio

## Discussion

COPD is associated with significant pulmonary arterial remodelling and stiffening; however, this stiffening is not associated with adverse right ventricular remodelling.

This is the first study examining PWV in COPD, demonstrating an elevated pulmonary PWV. The fact that an increased PWV was accompanied by a fall in PAT and a fall in the pulsatility provides further confirmatory evidence that significant central pulmonary vascular remodelling is present within the COPD cohort. In the current study we found that while there was a significant elevation in PWV, this was not associated with right ventricular hypertrophy.

In fact, despite the elevated arterial stiffness, the right ventricular remodelling evident in our study was that of a reduced preload rather than an increased afterload with evidence of smaller right ventricular volumes, smaller stroke volume and no significant increase in right ventricular mass. A similar pattern was observed in a small study of 24 COPD patients by Wells et al., where pulmonary arterial dilation was not associated with any increase in right ventricular mass despite being associated with a fall in right ventricular ejection fraction [26]. In the current work, both right and left ventricular volumes demonstrated a correlation with DLCO. It is well established that severe emphysema is associated with reduced cardiac volumes, thus our finding expands this observation of a correlation between emphysema and reduced cardiac filling into the milder end of the clinical spectrum [27]. This observation is also consistent with the findings of the MESA-COPD cohort, which also showed reduced right ventricular volumes with increasing emphysema burden as measured on CT in a predominantly non-severe cohort with COPD [28]. Thus the current study adds to the current literature that in a contemporary community population with COPD, the primary cardiac effects are those of emphysema induced preload reduction rather than that of cor pulmonale [28, 29].

As both the MESA-COPD cohort and the current study are predominantly within the milder spectrum of COPD, further work is required to determine if, as the disease progresses into the very severe end of the spectrum, a shift occurs from a preload driven phenotype into an afterload driven phenotype. This is an important clinical consideration as both require different management strategies with the former benefiting from volume reduction strategies [30], while the latter benefits from medications targeting the pulmonary vasculature [31]. Further examination in a more severe disease cohort is even more important as, if the same right ventricular pattern of preload remodelling is still present, it may provide a significant explanatory factor as to the continued failure of traditional pulmonary hypertension medications in a COPD cohort [32–35].

This is the first study to look at longitudinal changes in ventricular function in COPD. Due to this lack of prior work, the primary study outcome – change in right ventricular mass – was powered according to the placebo arms of randomised control trials in pulmonary hypertension. The rationale for this was that all the cardiac MRI studies at the time of the study design had described right ventricular hypertrophy, with the severity of this increasing with COPD severity [36–39]. However, as we have seen, we found a pattern of reduced ventricular volumes with no evidence of right ventricular hypertrophy. Our observed right ventricular mass of 39 g is also significantly lower than the mass of 74–102 g seen in the pulmonary hypertension interventional trials. Thus it is perhaps not surprising that at 1-year follow-up we detected no significant interval change in right ventricular mass. Given that the change in absolute RV mass only equated to an increase of 0.44 g, with a similarly small change in other ventricular metrics, this suggests that cardiac remodelling occurs slowly and can largely be considered stable over the usual time period used in interventional studies. As a result, we are unable to examine the longitudinal effects of pulmonary PWV on ventricular remodelling other than to say that if it is present it is likely small and beyond the power of this study to detect it.

There are several limitations to the current study. As previously mentioned, the current cohort were predominantly earlier stage COPD, although as this is the main subtype seen in the community this more accurately reflects the clinical environment rather than a study focussing on the severe end of the spectrum. Despite attempted matching, the COPD cohort were older and more obese, thus effects of these cannot be discounted. A small effect of age on pulmonary PWV have been previously described, but this is dwarfed by the 50 % difference in PWV we observed between the two cohorts, thus the effect is likely to be independent of this confounder [40]. In comparison, no such literature exists demonstrating an effect of obesity on pulmonary arterial stiffness and we observed no association within this cohort suggesting the bias of this confounder is limited. The study used multiple pairwise comparisons, and thus risk of type I error occurring has to be considered. However, where *p*-values were borderline and would have lost significance, the findings were congruent with the available literature in areas such as in the ventricular volumes. Finally, no invasive measures of pulmonary pressure were performed. The PAT, known to correlate well with mean pulmonary arterial pressure, was significantly lower in those with COPD, which would suggest elevated pressures; however, future invasive studies may be useful to evaluate the correlation between non-invasive PWV and both rest and exercise induced pulmonary hypertension in COPD.

While pulmonary vascular remodelling is present in COPD, cardiac morphology and function favour reduced preload rather than increased afterload. Treatment of obstructive lung disease may have greater effect on cardiac function than treatment of pulmonary vascular disease in most COPD patients.

## Electronic supplementary material

ESM 1 (DOCX 150 kb)

## Abbreviations

6MWT: 6-min walking test
BMI: Body mass index
BP: Blood pressure
CMR: Cardiac magnetic resonance
COPD: Chronic obstructive pulmonary disease
DLCO: Diffusing Capacity of the Lungs for Carbon Monoxide
ECG: Electrocardiogram
ECHO: Echocardiogram
FEF: Forced expiratory flow
FEV1: Forced expiratory volume in 1 s
FVC: Forced vital capacity
GOLD: Global Initiative for Chronic Obstructive Lung Disease
HR: Heart rate
HC: Healthy controls
ICS: Inhaled corticosteroid
KCO: Transfer factor for carbon monoxide
LABA: Long-acting beta-agonist
LAMA: Long-acting muscarinic antagonist
LV: Left ventricle
LVEDV: Left ventricle end-diastolic volume
LVEF: Left ventricle ejection fraction
LVESV: Left ventricle end-systolic volume
LVMVR: Light ventricular mass to volume ratio
LVM: Left ventricular mass
LVSV: Left ventricle stroke volume
mMRC: Modified British Medical Research Council
MPA: Main pulmonary artery
MRI: Magnetic resonance imaging
PA: Pulmonary artery
PAT: Pulmonary acceleration time
PC: Phase contrast
PFTs: Pulmonary function tests
PWV: Pulse wave velocity
RLV: Residual lung volume
RV: Right ventricle
RVEDV: Right ventricle end-diastolic volume
RVEF: Right ventricle ejection fraction
RVESV: Right ventricle end-systolic volume
RVH: Right ventricular hypertrophy
RVM: Right ventricular mass
RVMVR: Right ventricular mass to volume ratio
RVSV: Right ventricle stroke volume
SABA: Short-acting beta-agonist
SAMA: Short-acting muscarinic antagonist
SSFP: Steady-state free precession
TE: Time to echo
TLC: Total lung capacity
TR: Time to recovery
VENC: Velocity encoding
