# Supplementary material for: Pulmonary arterial stiffening in COPD and its implications for right ventricular remodelling
Source: Eur Radiol. 2018 Feb 27;28(8):3464–72. doi: 10.1007/s00330-018-5346-x (PMC6028842; doi:10.1007/s00330-018-5346-x)
Supplement: Supplementary file 1 — (DOCX 150 kb) [file 330_2018_5346_MOESM1_ESM.docx]

**Electronic Supplementary Material**

**Table S1:** Absolute agreement average measure intraclass correlation coefficients for repeated measures of right and left ventricular volumes and function.

| Ventricular metric | ICC | 95% CI | p |
| --- | --- | --- | --- |
| RVEDV | 0.99 | 0.96-0.99 | <0.001 |
| RVESV | 0.94 | 0.87-0.98 | <0.001 |
| RVSV | 0.96 | 0.91-0.99 | <0.001 |
| RVEF | 0.84 | 0.64-0.93 | <0.001 |
| RVM | 0.86 | 0.69-0.94 | <0.001 |

**Figure S1:** Bland-Altman plots of intra-observer differences between right ventricular measures.

**
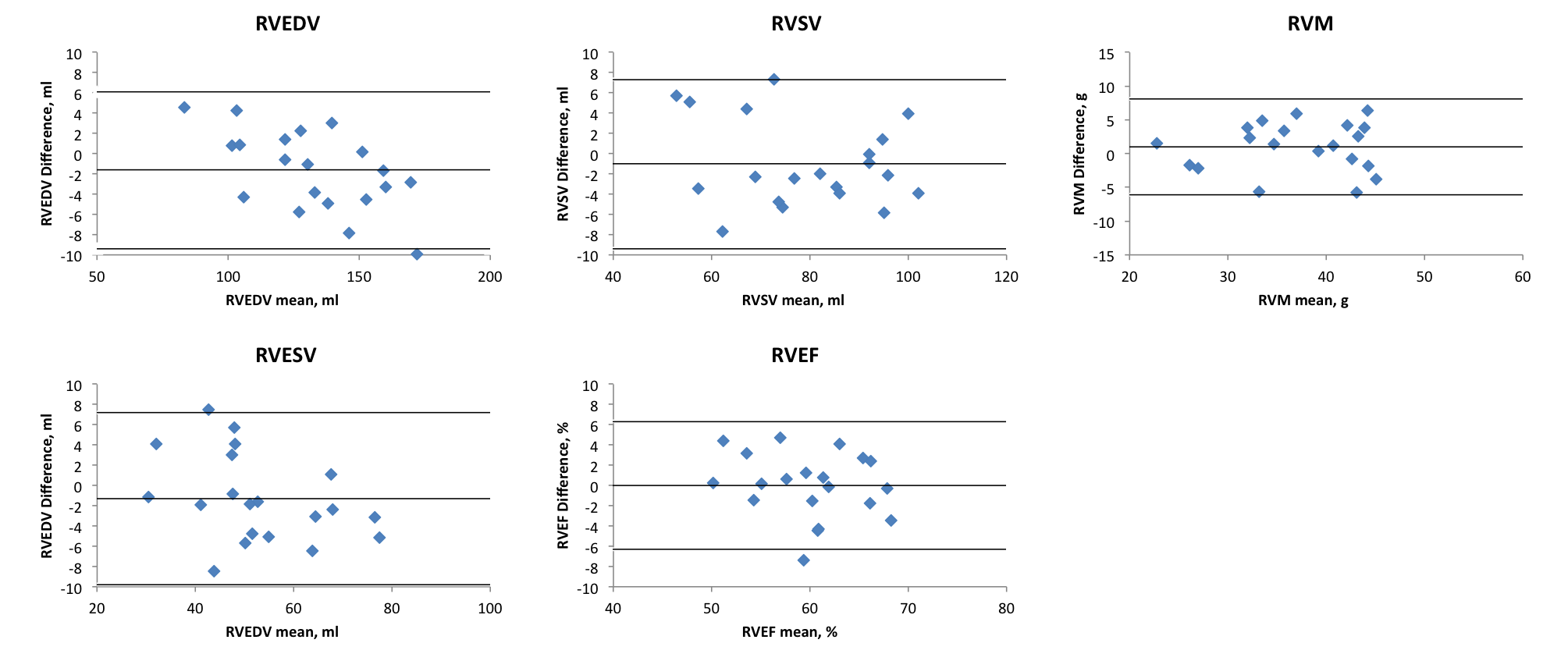
**

**Table S2: Comparison of those who only underwent baseline scan compared with those who completed both study visits.**

|  | **Baseline only cohort** | **Follow-up cohort** | **p** |
| --- | --- | --- | --- |
| N | 26 | 32 |  |
| Age | 66.1 ± 10.4 | 68.6 ± 8.2 | 0.31 |
| Sex (male) | 14 (54%) | 16 (50%) | 0.77 |
| BMI (kg/m^2^) | 26.4 ± 5.3 | 27.1 ± 5.3 | 0.63 |
| Heart rate (bpm) | 80.5 ± 21.7 | 69.1 ± 18.2 | **0.039** |
| Systolic BP (mmHg) | 130 ± 24 | 132 ± 17 | 0.74 |
| Diastolic BP (mmHg) | 76 ± 9 | 74 ± 8 | 0.39 |
| SpO2 | 95.9 ± 1.9 | 95.9 ± 1.7 | 0.98 |
| Smoking status | | | |
| Current smoker | 11 (42%) | 6 (19%) | 0.08 |
| Ex-smoker | 15 (58%) | 26 (81%) | 0.08 |
| Never smoker | 0 | 0 | 1 |
| Pack years | 44.0 ± 18.7 | 52.1 ± 27.3 | 0.20 |
| Medications | | | |
| SABA | 25 (96%) | 29 (91%) | 0.62 |
| SAMA | 1 (4%) | 0 | 0.45 |
| LABA | 4 (15%) | 4 (13%) | 1 |
| LAMA | 16 (62%) | 20 (77%) | 0.94 |
| ICS | 1 (4%) | 6 (19%) | 0.12 |
| LABA/ICS combo | 15 (58%) | 13 (41%) | 0.20 |
| Oral steroid | 1 (4%) | 3 (9%) | 0.62 |
| Antibiotics | 1 (4%) | 1 (3%) | 1 |
| Theophylline | 2 (8%) | 2 (6%) | 1 |
| Mucolytics | 4 (15%) | 5 (16%) | 1 |
| GOLD status | | | |
| I | 4 (15%) | 8 (25%) | 0.61 |
| II | 16 (62%) | 16 (50%) |  |
| III | 6 (23%) | 7 (22%) |  |
| IV | 0 | 1 (3%) |  |
| mMRC grade | | | |
| 0 | 2 (8%) | 3 (9%) | 0.17 |
| 1 | 10 (38%) | 16 (50%) |  |
| 2 | 9 (73%) | 5 (16%) |  |
| 3 | 3 (12%) | 8 (25%) |  |
| 4 | 2 (8%) | 0 |  |
| Pulmonary Function tests |  |  |  |
| FEV1, % predicted | 66.9 ± 22.7 | 65.5 ± 21.0 | 0.81 |
| FVC, % predicted | 99.2 ± 21.5 | 100.0 ± 19.6 | 0.87 |
| FEV1/FVC | 53.3 ± 10.2 | 51.2 ± 11.6 | 0.49 |
| FEF 25-75, % predicted | 25.5 ± 12.1 | 24.2 ± 12.5 | 0.70 |
| DLCO, % predicted | 61.2 ± 23.5 | 54.5 ± 16.7 | 0.22 |
| DLCO/VA, % predicted | 79.4 ± 23.9 | 71.1 ± 19.5 | 0.16 |
| RLV, % predicted | 176 ± 49 | 176 ± 56 | 0.97 |
| VC, % predicted | 98.9 ± 20.0 | 100.6 ± 17.4 | 0.74 |
| TLC, % predicted | 124.2 ± 16.6 | 124.8 ± 22.9 | 0.92 |
| RLV /TLC | 0.55 ± 0.11 | 0.55 ± 0.11 | 0.92 |
| 6MWT (m) | 428 ± 121 | 471 ± 97 | 0.14 |
| CMR |  |  |  |
| RVEDV (ml/m^1.7^) | 53.9 ± 12.8 | 53.4 ± 12.8 | 0.87 |
| RVESV (ml/m^1.7^) | 21.9 ± 8.1 | 21.7 ± 6.7 | 0.92 |
| RVSV (ml/m^1.7^) | 32.1 ± 7.0 | 31.7 ± 7.0 | 0.84 |
| RVEF (%) | 60.0 ± 7.0 | 59.7 ± 8.5 | 0.37 |
| RVM (g/m^1.7^) | 16.5 ± 3.5 | 16.3 ± 3.4 | 0.83 |
| RVMVR (g/ml) | 0.31 ± 0.07 | 0.31 ± 0.05 | 0.65 |
| LVEDV (ml/m^1.7^) | 57.0 ± 14.8 | 55.5 ± 9.4 | 0.66 |
| LVESV (ml/m^1.7^) | 24.5 ± 12.6 | 22.3 ± 5.6 | 0.42 |
| LVSV (ml/m^1.7^) | 32.5 ± 7.3 | 33.2 ± 6.3 | 0.72 |
| LVEF (%) | 58.5 ± 11.0 | 60.0 ± 6.0 | 0.53 |
| LVM (g/m^1.7^) | 44.1 ± 10.2 | 42.9 ± 8.8 | 0.63 |
| LVMVR (g/ml) | 0.79 ± 0.16 | 0.77 ± 0.11 | 0.59 |
| Pulmonary PWV | 2.97 ± 1.6 | 2.34 ± 0.97 | 0.09 |
| Pulmonary Pulsatility | 25.6 ± 7.7 | 24.1 ± 8.7 | 0.50 |
| Pulmonary acceleration time | 102.8 ± 22.8 | 105.1 ± 23.5 | 0.71 |
| Aortic PWV | 9.3 ± 3.1 | 8.2 ± 2.2 | 0.13 |

**Table S3: ANCOVA analysis of PWV, RVEDV and RVSV between those with COPD and the healthy controls.**

|  | Partial Eta Squared | F-test, P-value |
| --- | --- | --- |
| PWV | | |
| PWV | 0.05 | F=4.0, p=0.050 |
| Age | 0.03 | F=2.3, p=0.13 |
| BMI | 0.01 | F= 0.9, p=0.34 |
| Smoking pack years | 0.37 | F=43.5, p<0.001 |
| RVEDV | | |
| RVEDV | 0.06 | F=4.6, p=0.035 |
| Age | 0.03 | F=2.3, p=0.01 |
| BMI | 0.01 | F=1.0, p=0.33 |
| Smoking pack years | 0.38 | F=46.0, p<0.001 |
| RVSV | | |
| RVSV | 0.11 | F=4.6, p=0.035 |
| Age | 0.01 | F=0.7, p=0.42 |
| BMI | 0.02 | F=1.4, p=0.25 |
| Smoking pack years | 0.40 | F=49.6, p<0.001 |

RVEDV and RVSV are indexed to height^1.7

**Table S4: Change in demographics, pulmonary function tests and CMR parameters at 1 year follow-up.**

|  | **Baseline** | **1 year** | **Interval change (%)** | **P*** |
| --- | --- | --- | --- | --- |
| Age | 68.6 ± 8.2 | 69.9 ± 8.2 |  |  |
| BMI (kg/m^2^) | 27.1 ± 5.3 | 27.2 ± 5.2 | +0.2% | 0.82 |
| Heart rate (bpm) | 67.1 ± 11.2 | 68.2 ± 10.6 | +2% | 0.62 |
| Systolic BP (mmHg) | 133.4 ± 12.9 | 129.8 ± 18.2 | -3% | 0.19 |
| Diastolic BP (mmHg) | 75.8 ± 9.6 | 73.3 ± 9.6 | -1% | 0.18 |
| SpO2 | 95.9 ± 1.7 | 96.3 ± 2.2 | +0.4% | 0.20 |
| FEV1, % predicted | 65.5 ± 21.0 | 63.6 ± 20.2 | -3% | 0.24 |
| FVC, % predicted | 100 ± 20 | 100 ± 22 | 0% | 0.88 |
| FEV1/FVC | 51.3 ± 11.6 | 49.7 ± 9.9 | -3% | 0.05 |
| RVEDV (ml/m^1.7^) | 53.4 ± 9.6 | 52.1 ± 10.8 | -2% | 0.37 |
| RVESV (ml/m^1.7^) | 21.7 ± 6.7 | 21.8 ± 7.5 | +3% | 0.93 |
| RVSV (ml/m^1.7^) | 31.7 ± 7.0 | 30.4 ± 7.1 | -4% | 0.14 |
| RVEF (%) | 59.7 ± 8.5 | 58.6 ± 9.2 | -2% | 0.37 |
| RVM (g/m^1.7^) | 16.3 ± 3.4 | 16.4 ± 3.1 | 0% | 0.91 |
| RVMVR (g/ml) | 0.31 ± 0.05 | 0.32 ± 0.05 | +5% | 0.19 |
